# Supplementary material for: Arginine methylation of the DDX5 helicase RGG/RG motif by PRMT5 regulates resolution of RNA:DNA hybrids
Source: EMBO J. 2019 Jun 21;38(15):e100986. doi: 10.15252/embj.2018100986 (PMC6669924; doi:10.15252/embj.2018100986)
Supplement: Supplementary file 1 — Appendix [file EMBJ-38-e100986-s001.pdf]

TABLE OF CONTENT APPENDIX FIGURES AND TABLE

Appendix Figure S1. DDX5 siRNAs increase global RNA:DNA hybrids.

Appendix Figure S2. DDX5 deficiency leads to spontaneous DNA damage and hypersensitivity of U2OS cells to replication stress.

Appendix Figure S3. PRMT5 deficiency leads to spontaneous DNA damage.

Appendix Figure S4. The RGG/RG motif of DDX5 is required for DDX5 function in cellular R-loop repression.

Appendix Figure S5. Mutation of the arginine residues at the RGG motif does not affect DDX5 R-loop-unwinding activity.

Appendix Figure S6. Identification of DDX5-interacting proteins by SILAC MS/MS spectrometry.

Appendix Figure S7. XRN2 requires DDX5 for R-loop repression *in vitro*.

Appendix Figure S8. XRN2 is required for R-loop repression in cells.

Appendix Figure S9. RT-qPCR analysis of gene expression.

Appendix Table S1. Primer list.

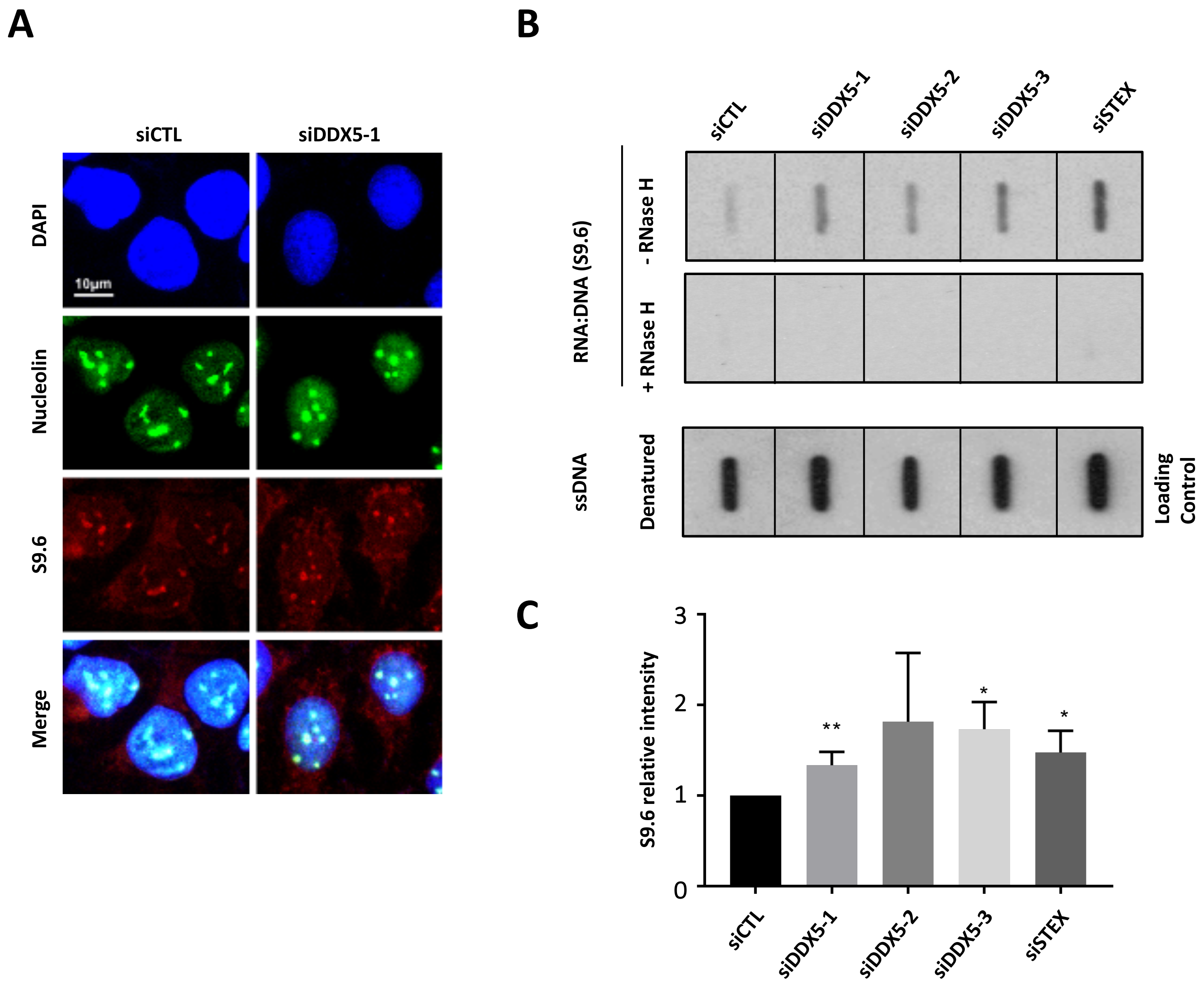

Appendix Figure S1. DDX5 siRNAs increase global RNA:DNA hybrids.

(A) The cells were subjected to immunofluorescence analysis with S9.6 and anti-nucleolin antibodies. A typical image was shown. The S9.6 signal in the nucleoplasm is measured as the total nuclear signal subtracting the nucleolar contribution detected as nucleolin-positive areas.

(B) Slot blot analysis of U2OS genomic DNA transfected with indicated siRNAs. The same amount of DNA extracted from siCTL, siDDX5, and siSTEX were left untreated, treated with RNase H (+) or denatured, as indicated. The membrane was subjected to immunoblotting with S9.6 antibodies or antibody recognizing single strand DNA (ssDNA) to verify equivalent DNA loading.

(C) The graphs show the relative quantification of panel B. Statistical significance was assessed using Student's t-test. *, $p < 0.05$; **, $p < 0.01$.

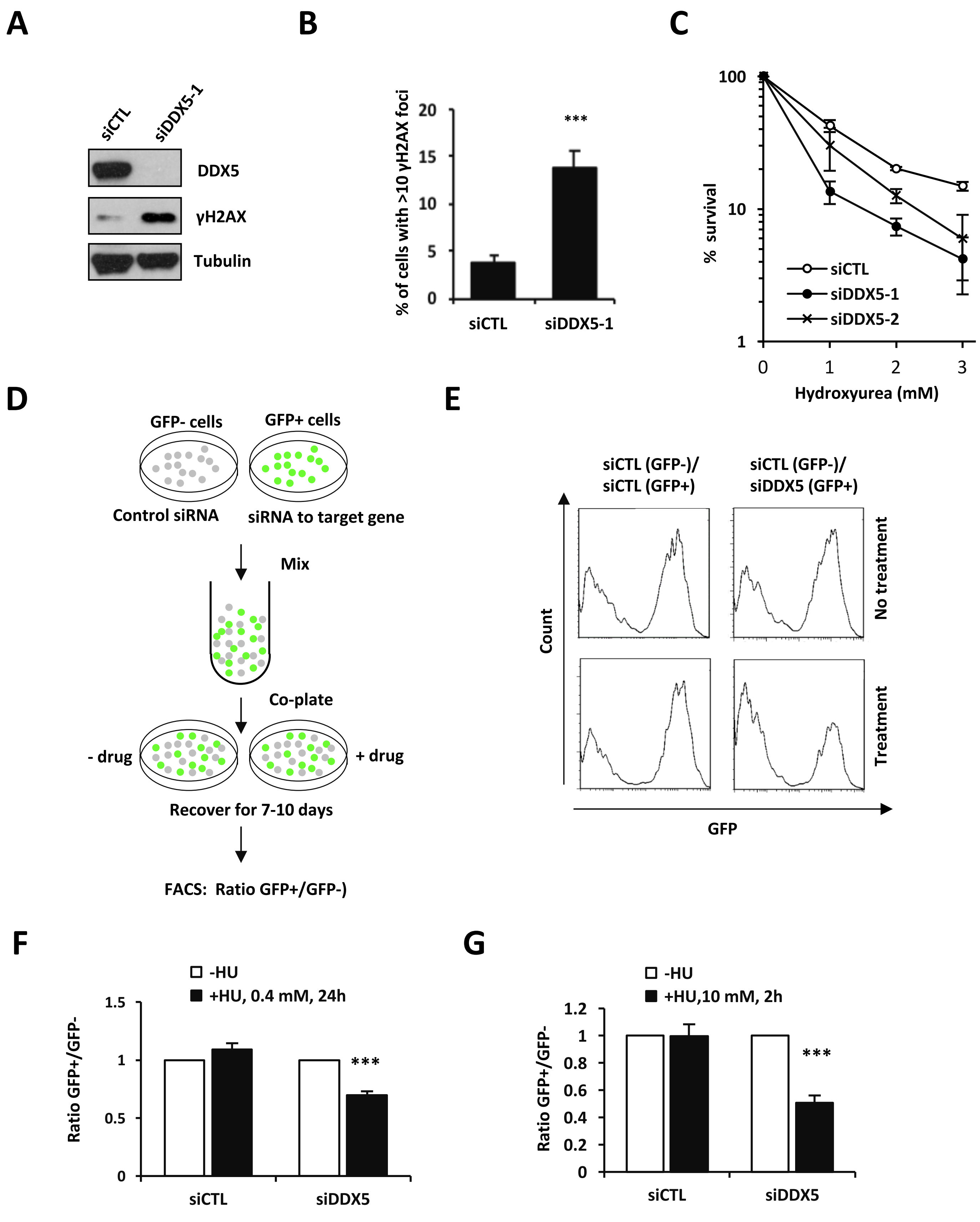
A
B
C
siCTL
siDDX5-1
DDX5
γH2AX
Tubulin
% of cells with >10 γH2AX foci
20
15
10
5
0
***
siCTL
siDDX5-1
100
10
1
% survival
siCTL
siDDX5-1
siDDX5-2
0
1
2
3
Hydroxyurea (mM)
D
GFP- cells
GFP+ cells
Control siRNA
siRNA to target gene
Mix
Co-plate
- drug
+ drug
Recover for 7-10 days
FACS: Ratio GFP+/GFP-)
E
siCTL (GFP-)/
siCTL (GFP+)
siCTL (GFP-)/
siDDX5 (GFP+)
Count
No treatment
Treatment
GFP
F
-HU
+HU, 0.4 mM, 24h
Ratio GFP+/GFP-
1.5
1
0.5
0
***
siCTL
siDDX5
G
-HU
+HU,10 mM, 2h
Ratio GFP+/GFP-
1.2
1
0.8
0.6
0.4
0.2
0
***
siCTL
siDDX5

Appendix Figure S2. DDX5 deficiency leads to spontaneous DNA damage and hypersensitivity of U2OS cells to replication stress.
(A) Immunoblotting analysis of total protein extracts derived from U2OS cells transfected with siCTL or siDDX5-1. (B) Foci quantification results after immunofluorescence with anti-γH2AX antibody of U2OS cells transfected with control (siCTL) or siDDX5-1. The cells with >10 foci were counted and expressed as a percentage of total cells. The graph shows the average and standard error of the mean (S.E.M.) from three independent experiments where >30 images were acquired containing at least 300 cells per condition. (C) Colony survival analysis of U2OS cells transfected with siDDX5 or control siRNAs. Cells were treated with different dosage of hydroxyurea (HU) for 20 h. Colony survival analysis was performed as described in the "Materials and Methods" section. The graph shows the average and standard error of the mean (SEM) from four independent experiments. (D) Illustration of FACS-based cell survival analysis. Wild type U2OS and U2OS stably expressing transfected GFP were transfected with siCTL and siDDX5. The cells were then harvested two days after transfection and mixed in an approximately 1:1 ratio. These cells were plated and exposed to hydroxyurea. Then FACS analysis was performed following seven to ten days of recovery.

(E) A FACS profile shows DDX5 deficiency leads to a decrease of GFP+ cell population and an increase of GFP- cell population in the hydroxyurea-treated cells, compared to untreated cells.

(F and G) Cell survival following treatment with 10 mM hydroxyurea (HU) for 2 h or 0.4 mM 24 h. Data are plotted as a ratio in GFP+ to GFP- cells normalized to untreated control. The graphs show the average and standard error of the mean (SEM) from four independent experiments. Statistical significance was assessed using Student's t-test; ***, $p < 0.001$.

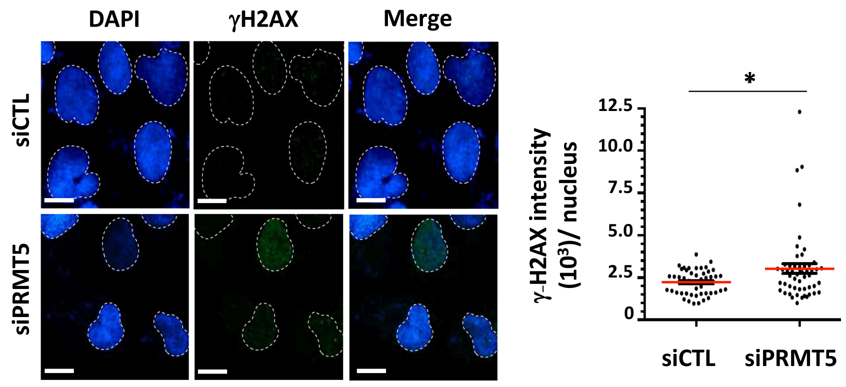

Appendix Figure S3. PRMT5 deficiency leads to spontaneous DNA damage.
U2OS cells were transfected with control or siPRMT5 siRNA. 72 hours later the cells were stained with anti-γH2AX (green) antibody and DAPI, and then visualized by indirect immunofluorescence using Zeiss LSM800 confocal system. A typical image was shown for each condition. Intensity of nuclear staining was measured in at least 50 cells chosen randomly from at least 13 different fields per condition. Each dot in the scatter plot represents the average intensity per nucleus, and the bar represents the overall average intensity per condition. Statistical significance was assessed using Student's t-test. *: $p < 0.05$.

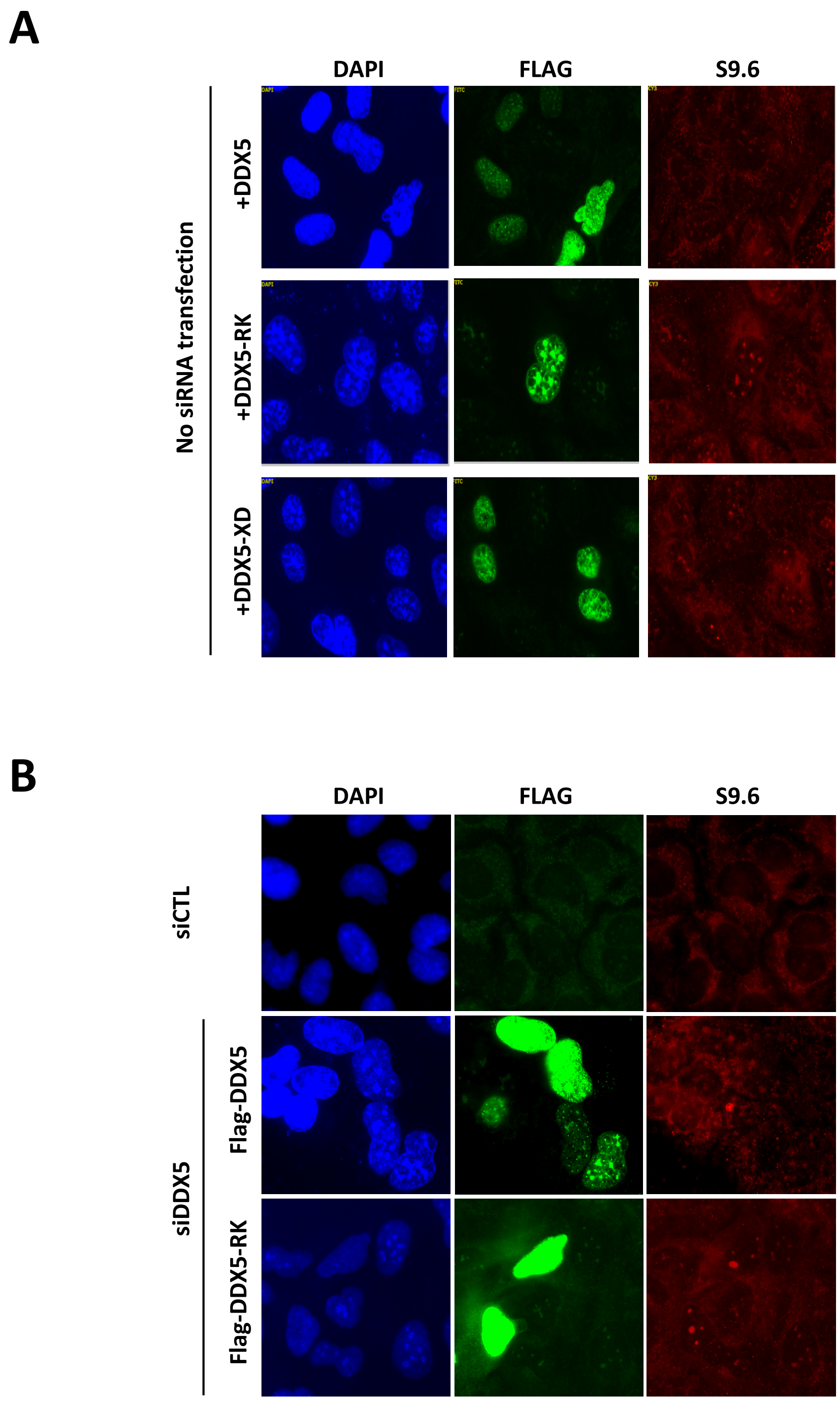

Appendix Figure S4. The RGG/RG motif of DDX5 is required for DDX5 function in cellular R-loop repression.

U2OS cells were transfected with indicated siRNAs and plasmid DNAs and subjected to immunofluorescence analysis with S9.6 and anti-Flag antibodies. A typical image was shown. The intensity of S9.6 image from various number of Flag-positive and negative cells was quantified and presented in Figure 5.

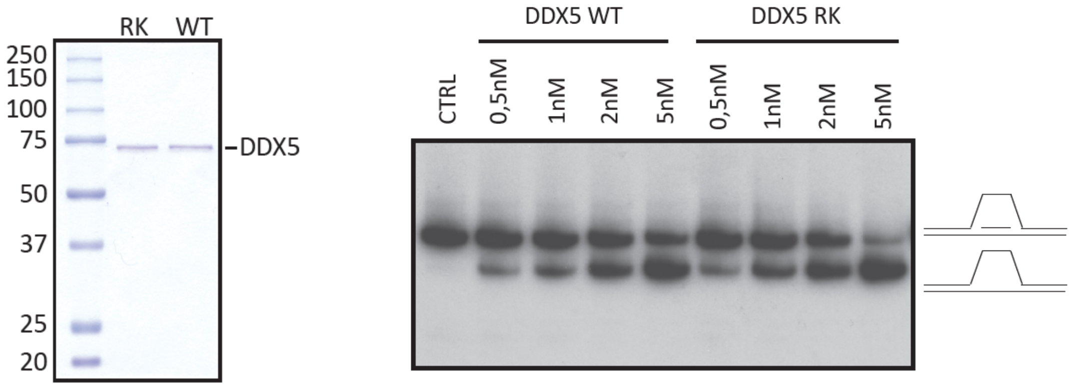

Appendix Figure S5. Mutation of the arginine residues at the RGG motif does not affect DDX5 R-loop-unwinding activity.

(A) Coomassie Blue staining of recombinant human DDX5 and its RK mutant (replacement of the five arginine residues at the RGG motif by lysine) purified in Sf9 insect cells. M denotes the molecular mass markers in kDa.

(B) R-loop unwinding assay in presence of increasing DDX5.

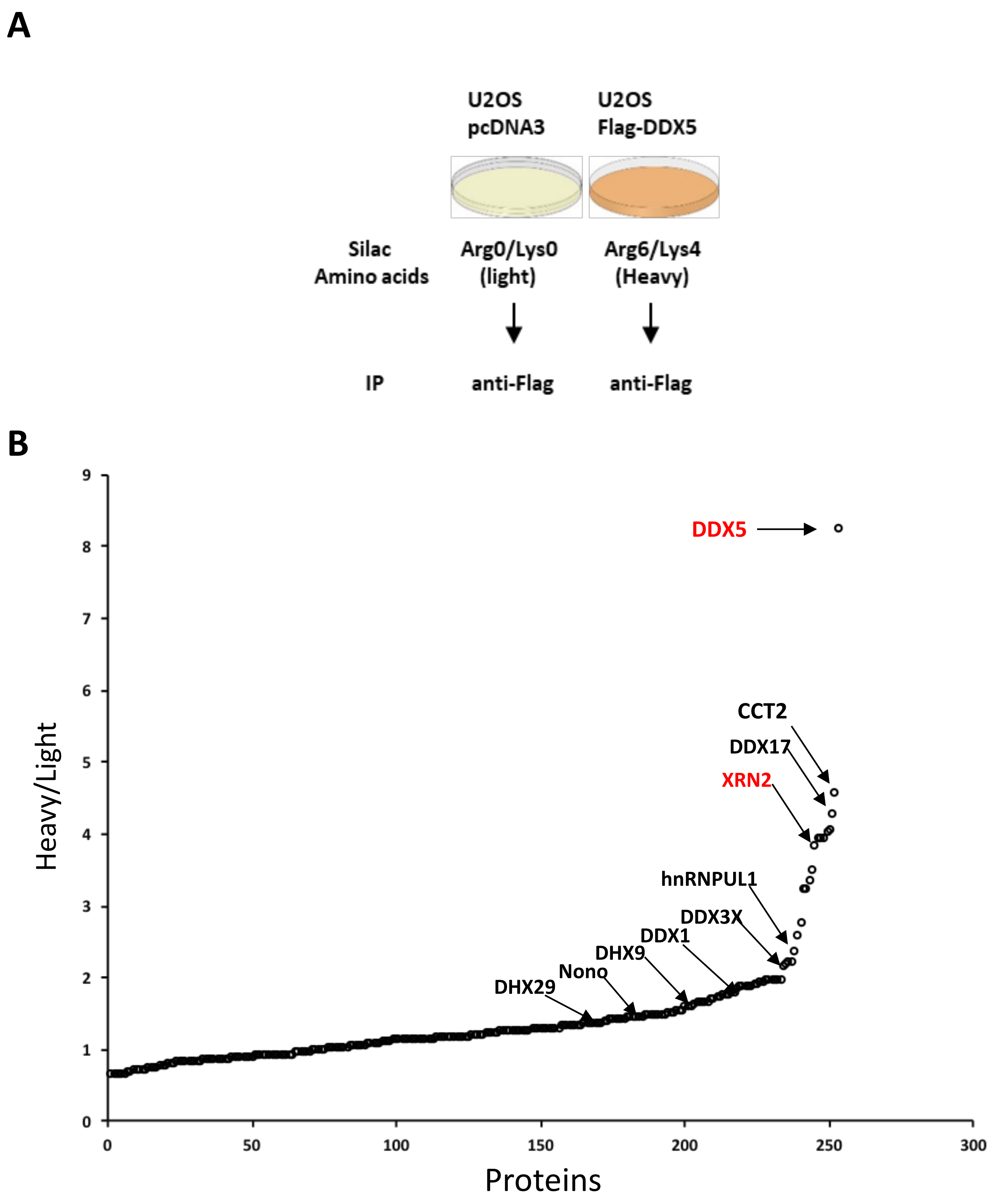

Appendix Figure S6. Identification of DDX5-interacting proteins by SILAC MS/MS spectrometry.

(A) U2OS cells with stably or transiently transfected Flag-DDX5 or empty vector were subjected to SILAC analysis as described in the "Materials and Methods" section.

(B) The identified proteins, including XRN2 with a heavy/light signal ratio were presented. (XRN2 ratio is 3.92).

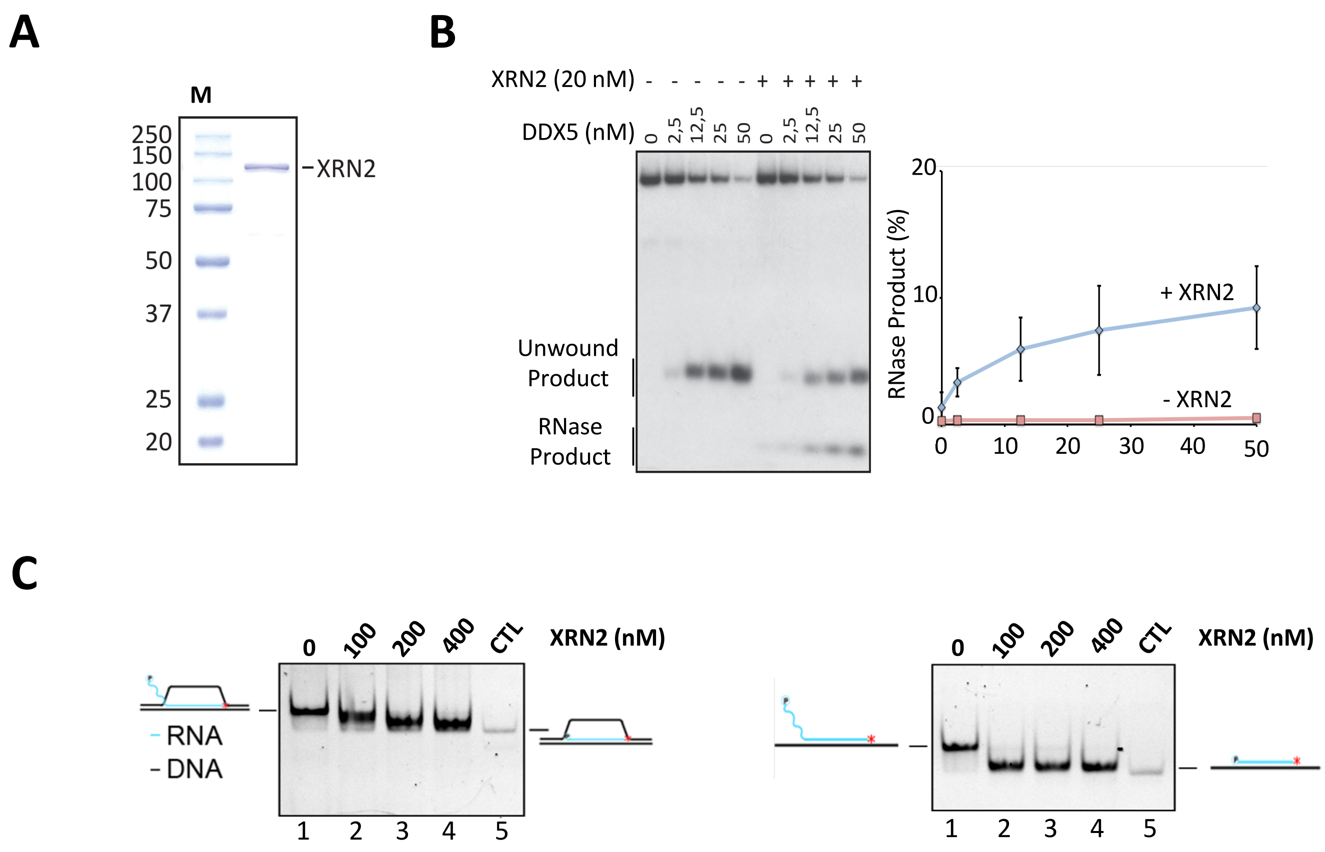

Appendix Figure S7. XRN2 requires DDX5 for R-loop repression *in vitro*.

(A) Coomassie Blue staining of recombinant human XRN2 purified from bacteria. M indicates the molecular mass markers in kDa.

(B) R-loop unwinding and RNA exonuclease activity analysis. A typical image is shown on the left. The data was analyzed as percentage of RNase product (right panel). The graph shows the average and S.E.M. n=4.

(C) XRN2 does not degrade RNA/DNA duplex on a 5' overhang R-loop substrate (left) or a 5' overhang RNA/DNA duplex.

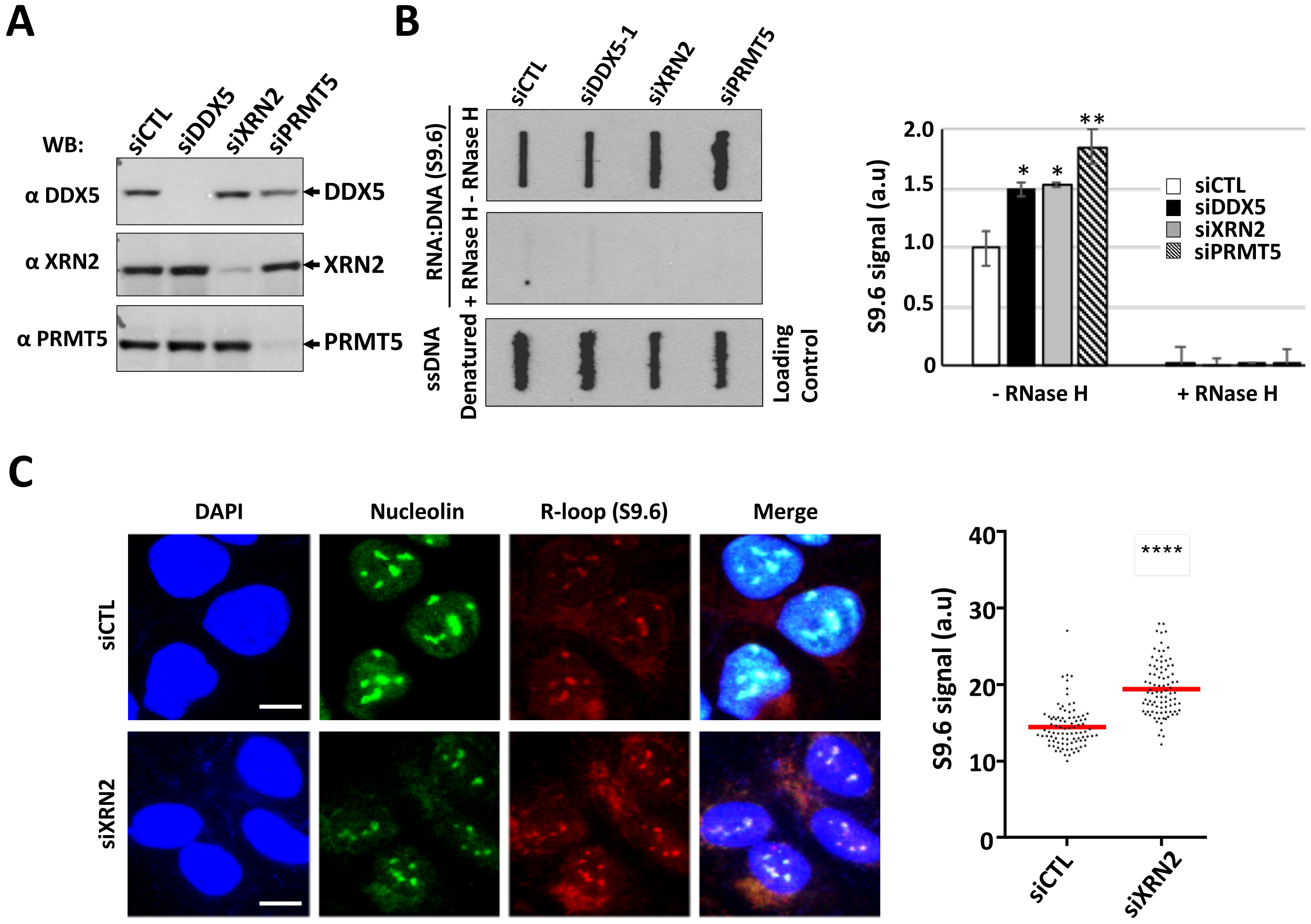

Appendix Figure S8. XRN2 is required for R-loop repression in cells.
(A) Western blot analysis of U2OS cells transfected with indicated siRNAs.
(B) Slot blot analysis of genomic DNA. The same amount of DNA extracted from siCTL, siDDX5, siXRN2 and siPRMT5 transfected U2OS cells were left untreated, treated with RNase H (+) or denatured, as indicated. The membrane was subjected to immunoblotting with S9.6 antibodies or antibody recognizing single strand DNA (ssDNA) to verify equivalent DNA loading. The graph shows the relative quantification and S.E.M from three independent experiments. Statistical significance was assessed using Student's t-test. *: $p < 0.05$; **: $p < 0.01$.
(C) The U2OS cells were subjected to immunofluorescence analysis with S9.6 and anti-nucleolin antibodies. A typical image was shown. The S9.6 signal in the nucleoplasm is measured as the total nuclear signal subtracting the nucleolar contribution detected as nucleolin-positive areas. The graph shows an average and S.E.M from 3 independent experiments performed in triplicate. Statistical significance was assessed using one-way ANOVA t-test. ****: $p < 0.0001$.

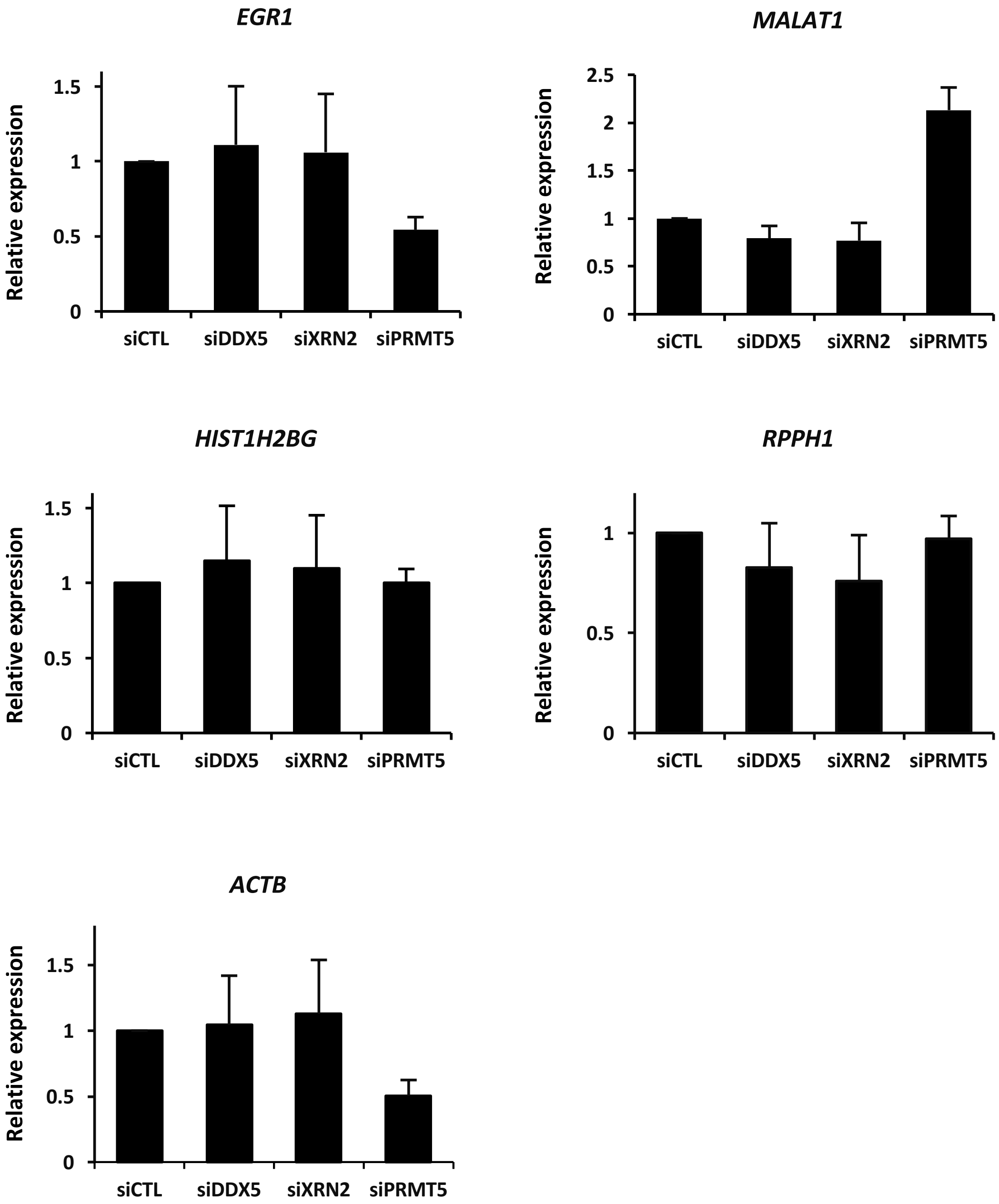

Appendix Figure S9. RT-qPCR analysis of gene expression.
U2OS cells were transfected with indicated siRNAs and the total RNA was extracted and subjected reverse transcription (RT) and qPCR analysis. GAPDH was used to normalize gene expression.

Appendix Table S1. Primer list.

Name	Sequence	Experiment
EGR1	F: TTCGGATTCCCGCAGTGT<br>R: TCACTTTCCCCCCTTTATCCA	DRIP
HIST1H2BG	F: TGTGACCAAGGCGCAGAAGA<br>R: GAGCGCTTGTTGTAGTGGGC	DRIP
RPPH1	F: GTGCGTCCTGTCACTCCACT<br>R: TTCCAAGCTCCGGCAAAGGA	DRIP & RT-qPCR
MALAT1	F: ACGCAGGGAGAATTGCGTCA<br>R: CCTTCCCGTACTTCTGTCTTCCA	DRIP & RT-qPCR
EGR1	F: GGTCAGTGGCCTAGTGAGC<br>R: GTGCCGCTGAGTAAATGGGA	RT-qPCR
HIST1H2BG	F: CTCCTGCTCCGAAGAAGGG<br>R: AGTAGCTCTCCTTACGACTGC	RT-qPCR
β-Actin	F: CATGTACGTTGCTATCCAGGC<br>R: CTCCTTAATGTCACGCACGAT	RT-qPCR
β-Actin Prom	F: ctcaatctcgctctcgctct<br>R: ctcgagccataaaaggcaac	DRIP & ChIP
β-Actin Region B	F: caactgggacgacatggagaa a<br>R: gagtcctacggaaaacggcaga	DRIP & ChIP
β-Actin Poly A	F: tgtacactgacttgagaccag t<br>R: aagcaggaacagagacctgacc	DRIP & ChIP
β-Actin Region D	F: taggcttaggagaggccgcaat<br>R: gtccaggagcctgggtatctcc	DRIP & ChIP
PRMT7	F: aattgcccttgaatgcgagaccct<br>R: tcacccaaggccaaactccaaaca	DRIP & ChIP
LINC01346 F	F: tcgtatgttgctggggatgacgtg<br>R: tcccactgtgcactaagttcaccc	DRIP & ChIP
NFKBIL2	F: gtctatgtcatccaccacgcccag<br>R: gtctggggatgaaagcagggtgag	DRIP & ChIP
SLC25A3	F: GTGTTCAAGCTATGGAGCCTGCC<br>R: gtgggcctcttgggttttgagga	DRIP & ChIP
JuN	F: CGACCCTCCCAGAGTTCCATGA<br>R: TGCAAGCAGAAACATGAAGGGGT	DRIP & ChIP
EEF1A1	F: TTGTTCCCACTGGAAGCAGGAATG<br>R: TGGTGCTCAAGCCACAGTTGTCT	DRIP & ChIP
