# Supplementary material for: Arginine methylation of the DDX5 helicase RGG/RG motif by PRMT5 regulates resolution of RNA:DNA hybrids
Source: EMBO J. 2019 Jun 21;38(15):e100986. doi: 10.15252/embj.2018100986 (PMC6669924; doi:10.15252/embj.2018100986)
Supplement: Supplementary file 4 — Source Data for Appendix [file EMBJ-38-e100986-s004.pdf]

Mersaoui et al., Figure S1

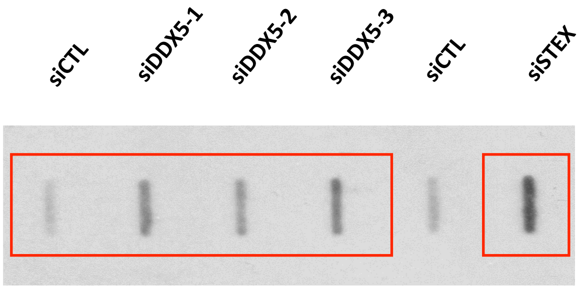

Figure S1 S9.6 Blot

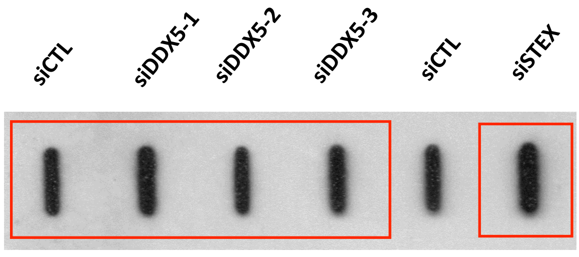

Figure S1 ssDNA Blot

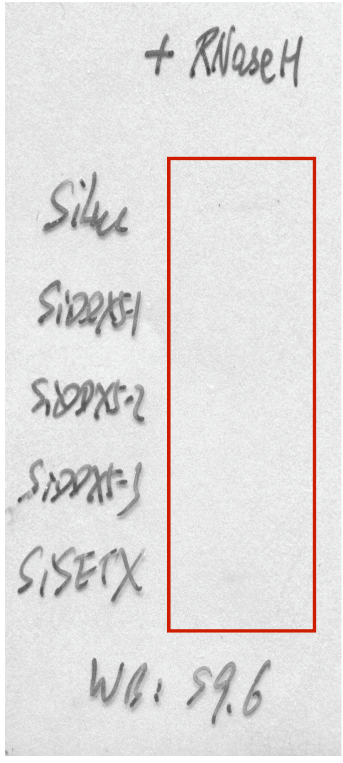

Figure S1 + RNaseH

Mersaoui et al., Figure S2

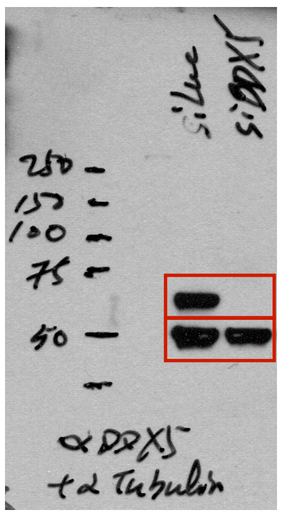

Figure S2A,
upper panel and
Lower panel

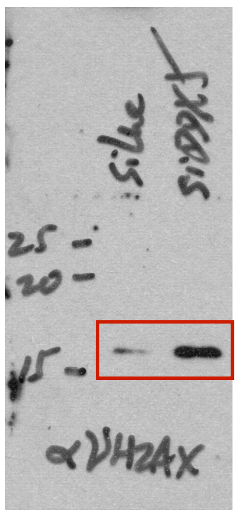

Figure S2A,
middle panel

Mersaoui et al., Figure S5

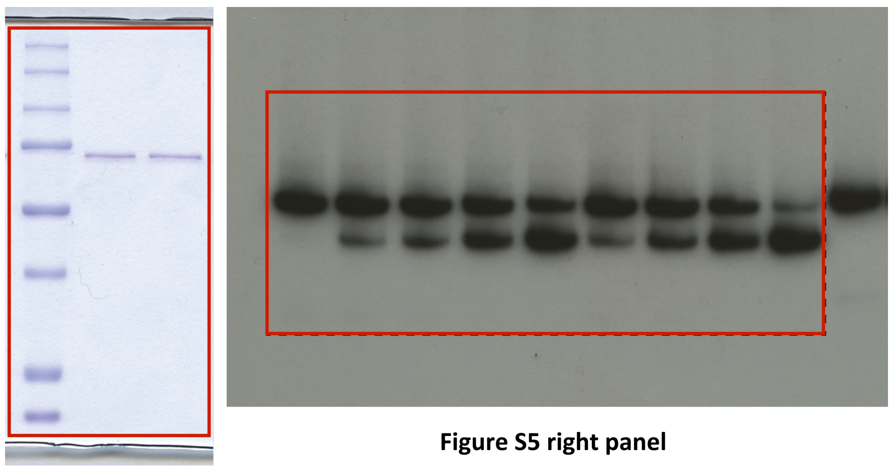

Figure S5 right panel

Figure S5 left panel

Mersaoui et al., Figure S7

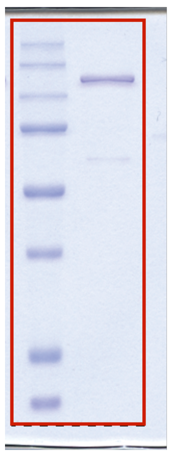

Figure S7A

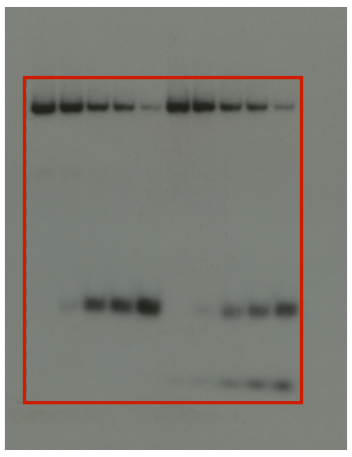

Figure S7B

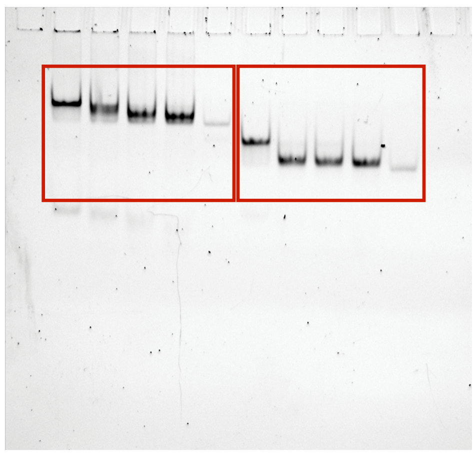

Figure S7C

Mersaoui et al., Figure S8

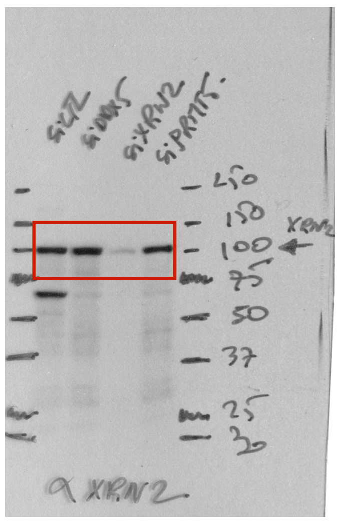

Figure S8A XRN2 blot

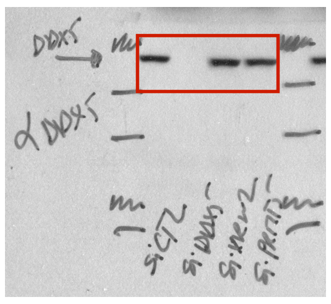

Figure S8A DDX5 blot

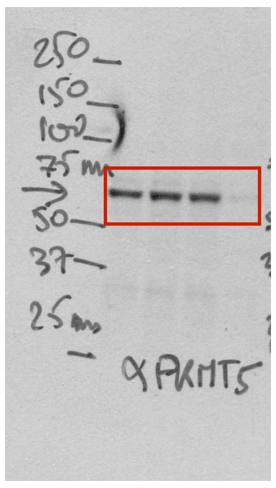

Figure S8A PRMT5 blot

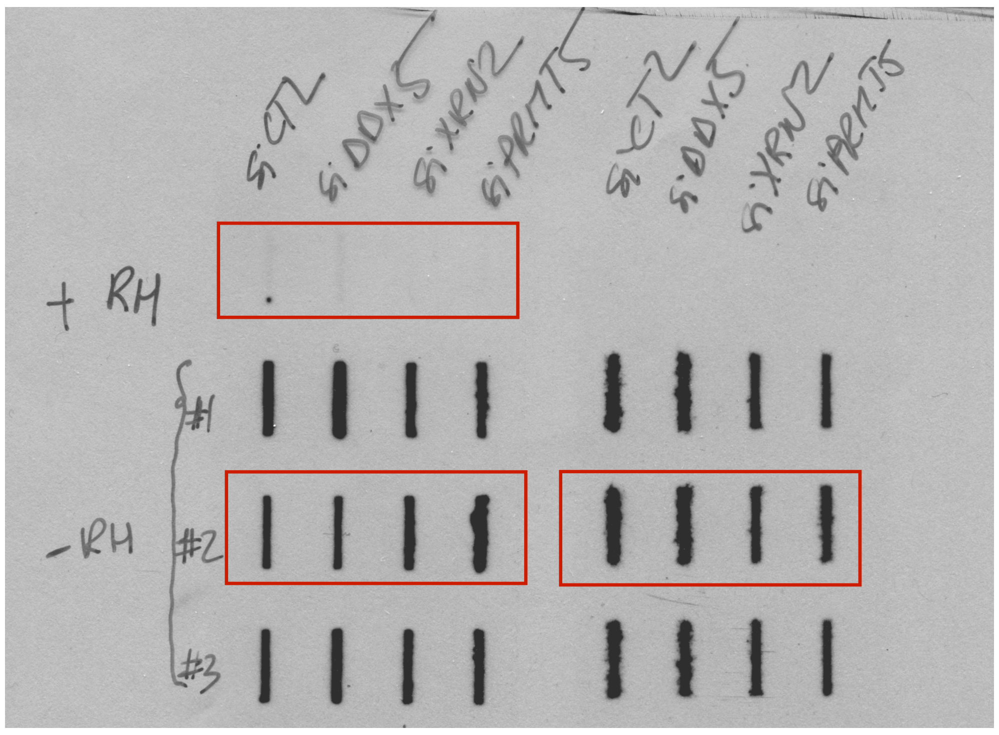

S9.6 ssDNA

Figure S8B PRMT5 blot
