# Supplementary figures and images for: Arginine methylation of the DDX5 helicase RGG/RG motif by PRMT5 regulates resolution of RNA:DNA hybrids
Source: EMBO J. 2019 Jun 21;38(15):e100986. doi: 10.15252/embj.2018100986 (PMC6669924; doi:10.15252/embj.2018100986)

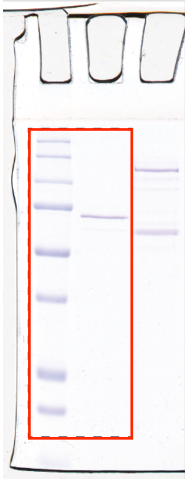

Figure 1A

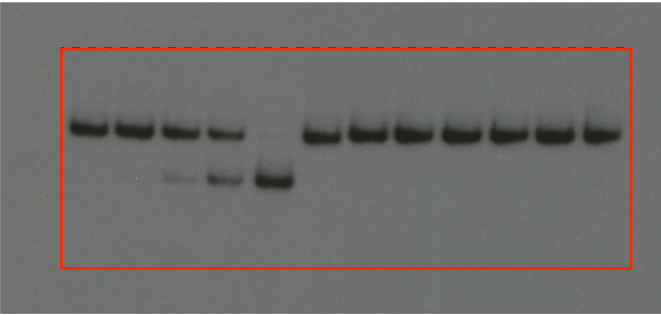

Figure 1B

Supplement: Supplementary file 6 — Source Data for Figure 1 [file EMBJ-38-e100986-s005.pdf]

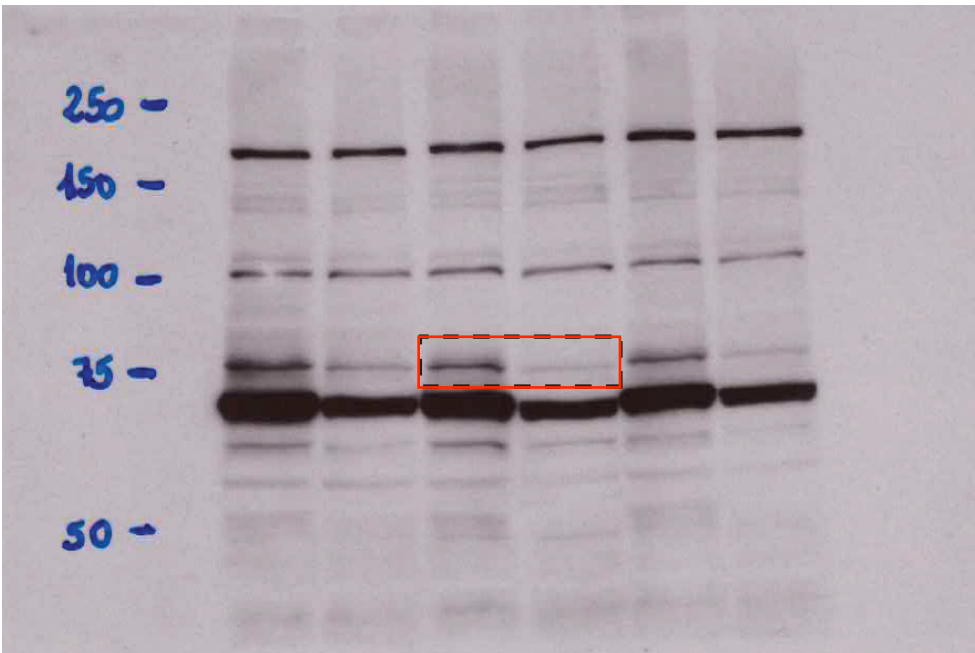

— siPRMT5

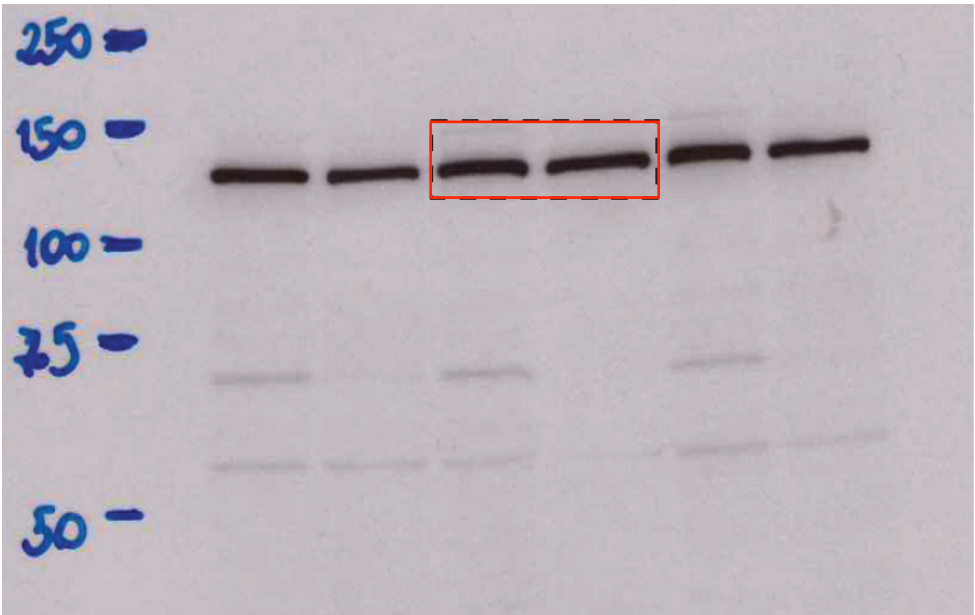

— Vinculin for siPRMT5

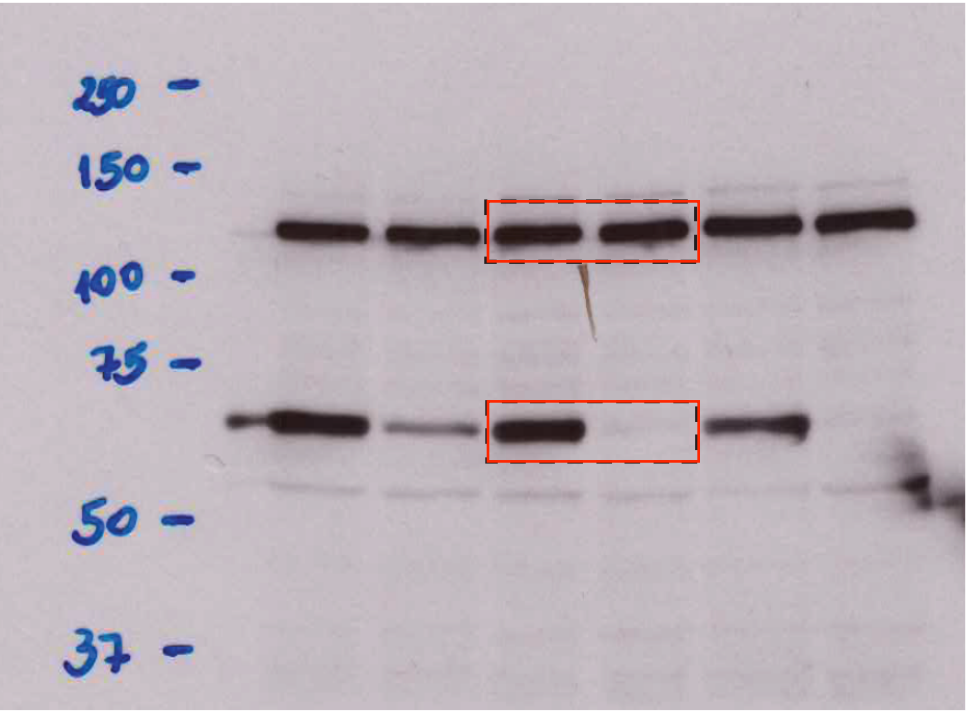

— Vinculin for siDDX5

— siDDX5

Supplement: Supplementary file 8 — Source Data for Figure 3 [file EMBJ-38-e100986-s007.pdf]

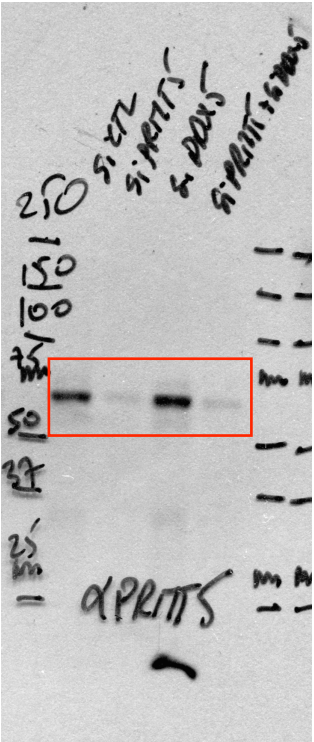

Figure 4B, PRMT5 blot

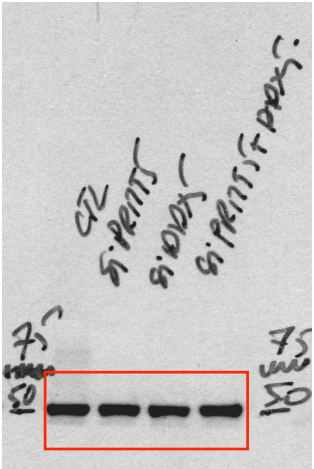

Figure 4B, Tubulin blot

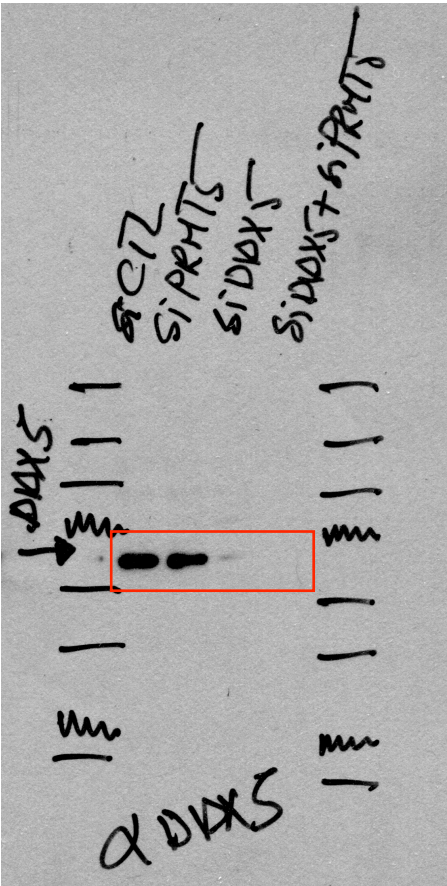

Figure 4B, DDX5 blot

Supplement: Supplementary file 9 — Source Data for Figure 4 [file EMBJ-38-e100986-s008.pdf]
