# Supplementary material for: Arginine methylation of the DDX5 helicase RGG/RG motif by PRMT5 regulates resolution of RNA:DNA hybrids
Source: EMBO J. 2019 Jun 21;38(15):e100986. doi: 10.15252/embj.2018100986 (PMC6669924; doi:10.15252/embj.2018100986)
Supplement: Supplementary file 7 — Source Data for Figure 2 [file EMBJ-38-e100986-s006.pdf]

Mersaoui et al., Figure 2

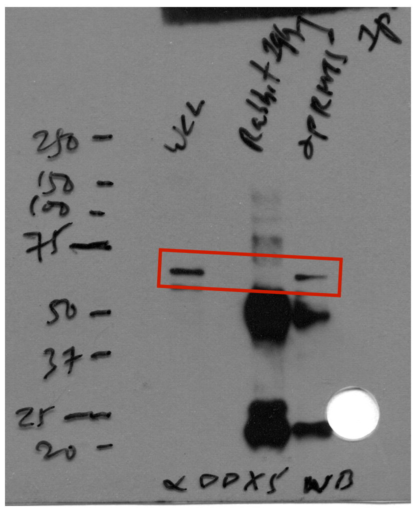

Figure 2A, upper panel

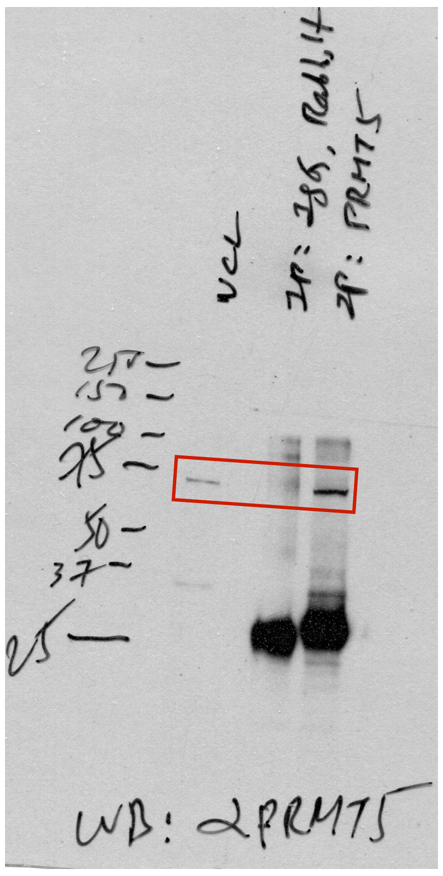

Figure 2A, lower panel

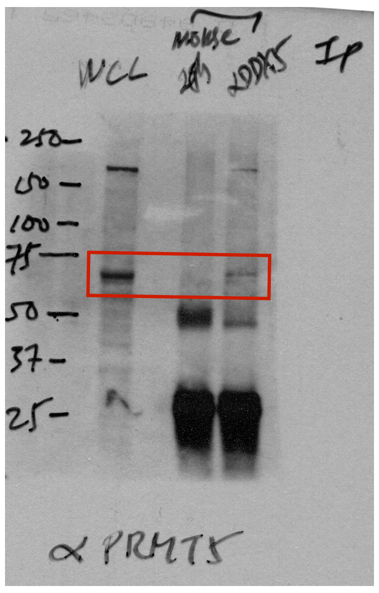

Figure 2B, upper panel

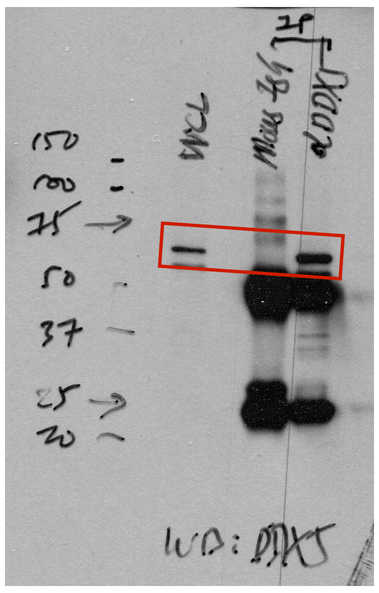

Figure 2B, lower panel

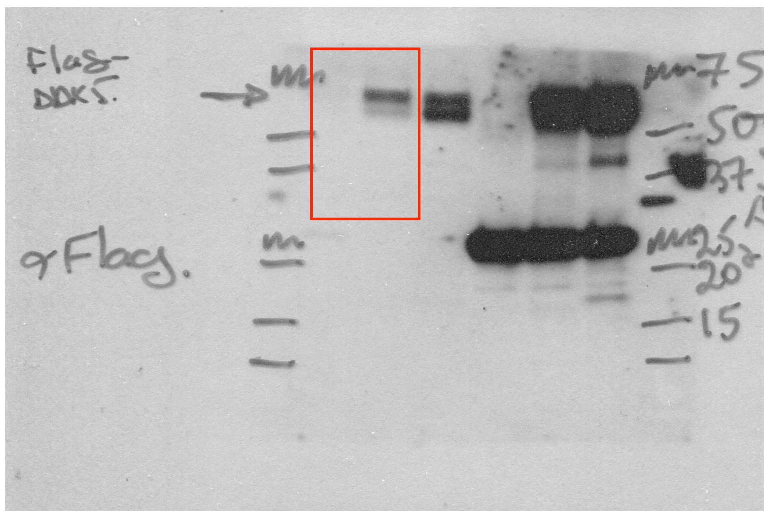

Figure 2D, Flag Blot for WCE

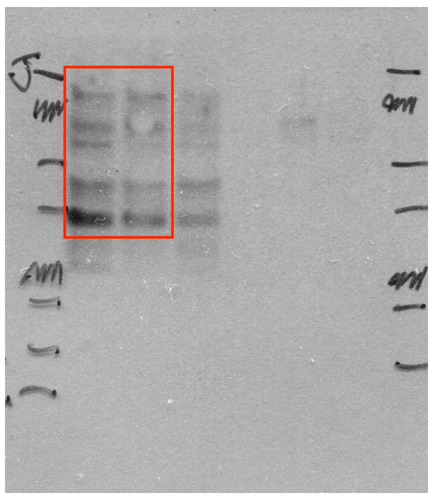

Figure 2D, MMA Blot for WCE

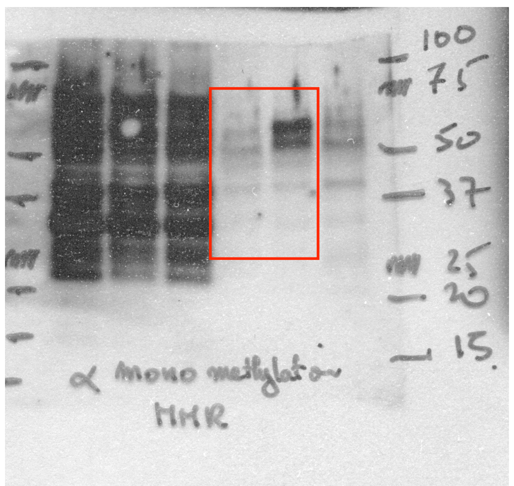

Figure 2D, MMA Blot for IP Flag

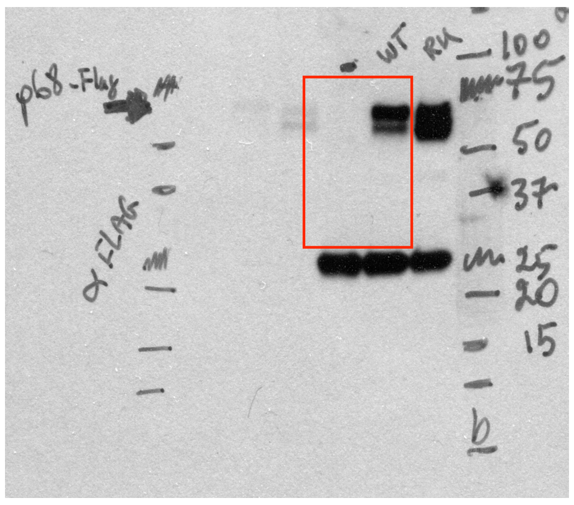

Figure 2D, Flag Blot for IP Flag

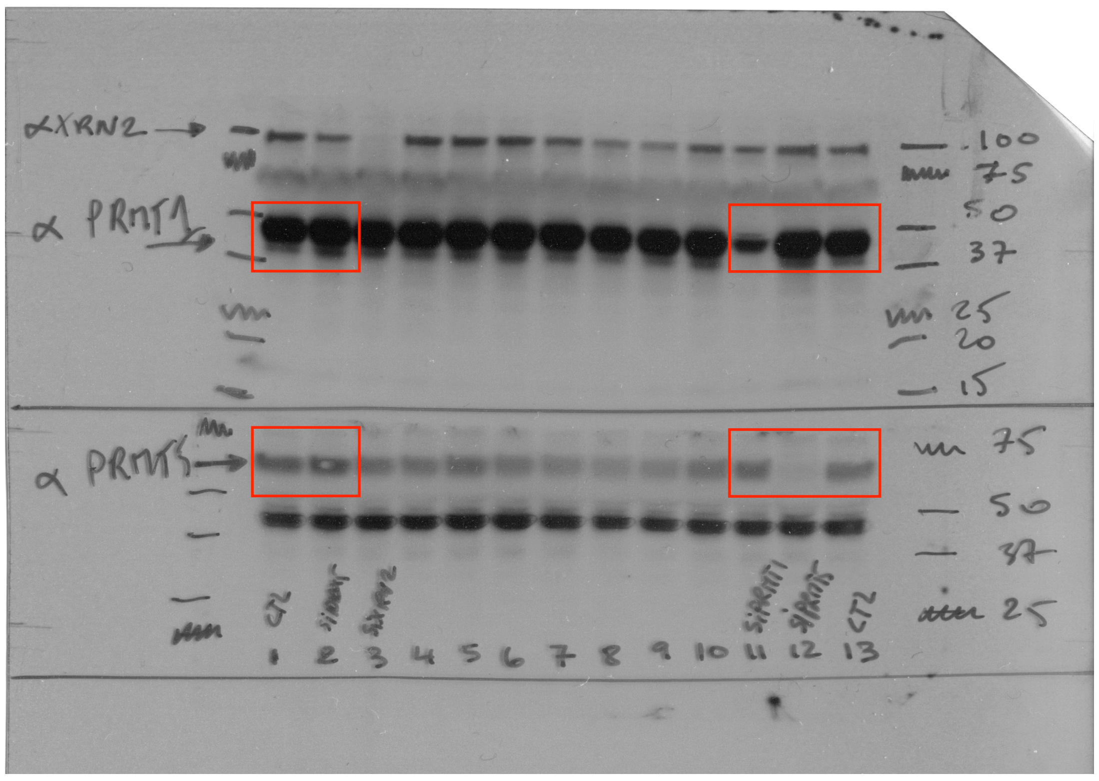

Figure 2E: PRMT1 and PRMT5 blots

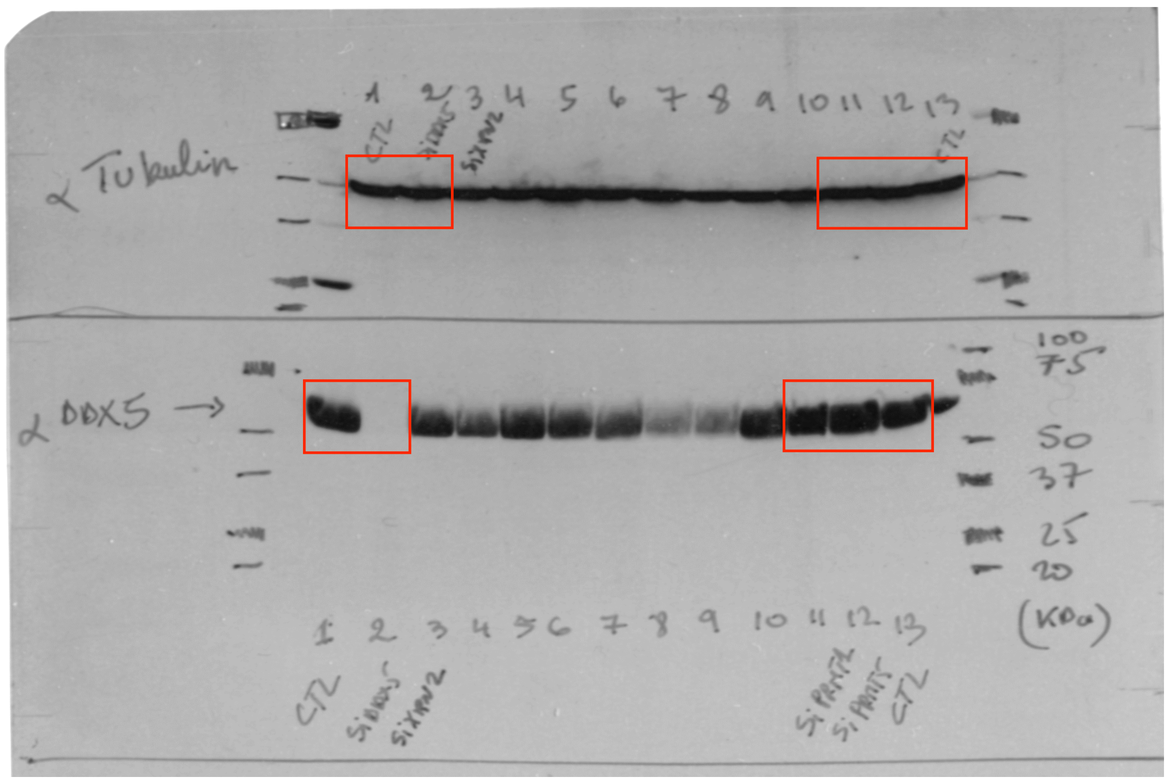

Figure 2E DDX5 and Tubulin Blots
