# Supplementary material for: Arginine methylation of the DDX5 helicase RGG/RG motif by PRMT5 regulates resolution of RNA:DNA hybrids
Source: EMBO J. 2019 Jun 21;38(15):e100986. doi: 10.15252/embj.2018100986 (PMC6669924; doi:10.15252/embj.2018100986)
Supplement: Supplementary file 10 — Source Data for Figure 5 [file EMBJ-38-e100986-s009.pdf]

Mersaoui et al., Figure 5

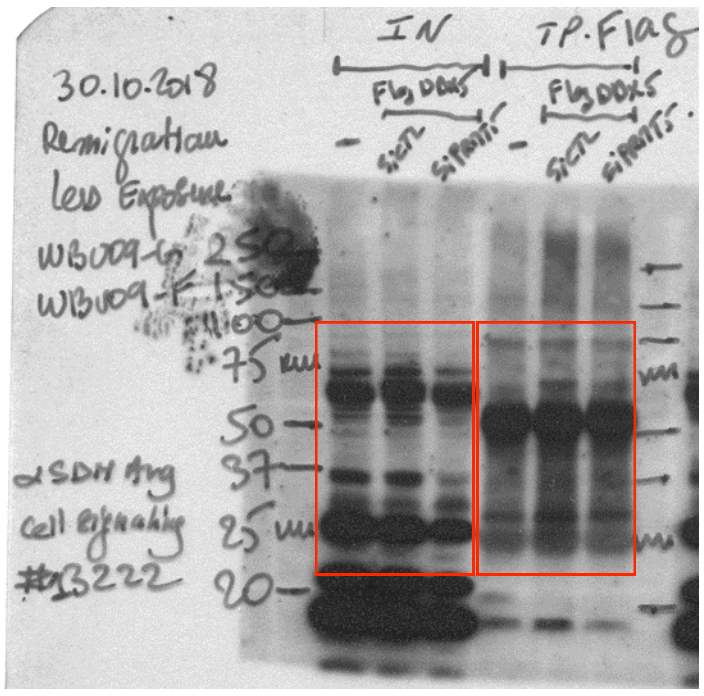

Figure 5A SDMA blot

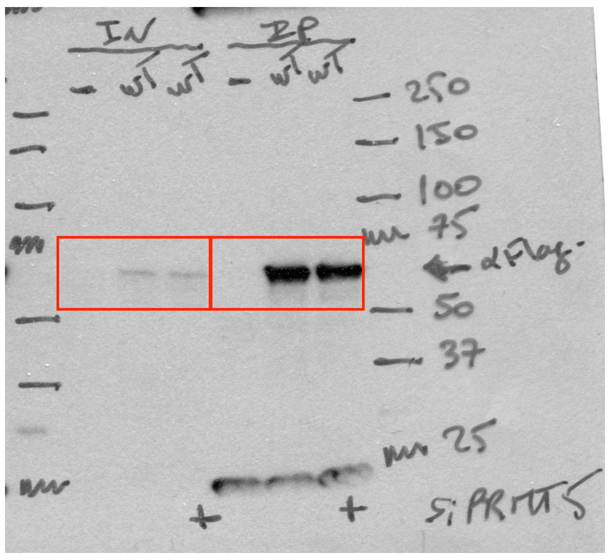

Figure 5A Flag blot

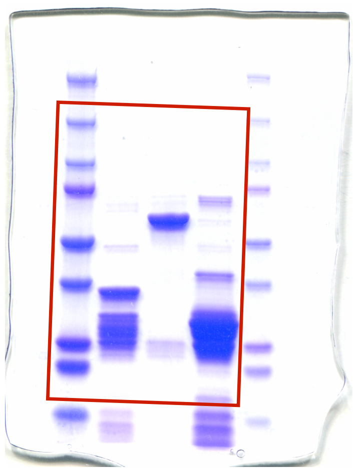

Figure 5D, left panel

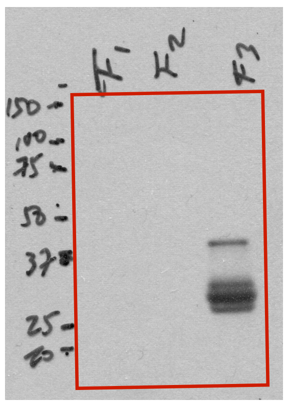

Figure 5D, right panel

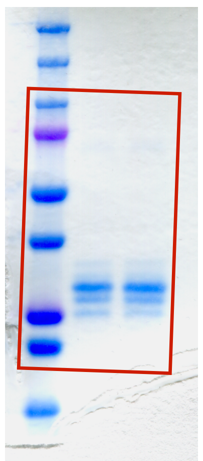

Figure 5E, left panel

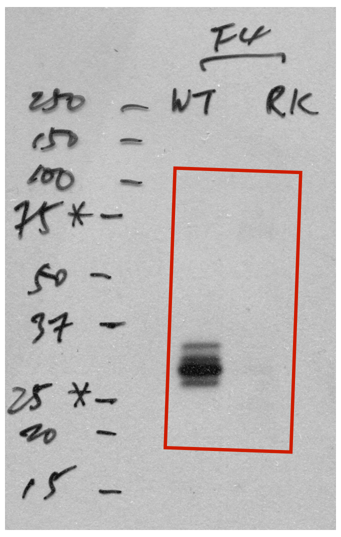

Figure 5E, right panel
