# Supplementary material for: Arginine methylation of the DDX5 helicase RGG/RG motif by PRMT5 regulates resolution of RNA:DNA hybrids
Source: EMBO J. 2019 Jun 21;38(15):e100986. doi: 10.15252/embj.2018100986 (PMC6669924; doi:10.15252/embj.2018100986)
Supplement: Supplementary file 11 — Source Data for Figure 6 [file EMBJ-38-e100986-s010.pdf]

Mersaoui et al., Figure 6

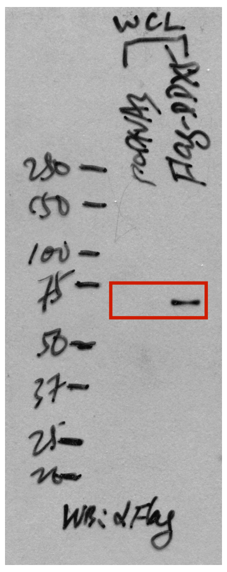

Figure 6A,
left, upper panel

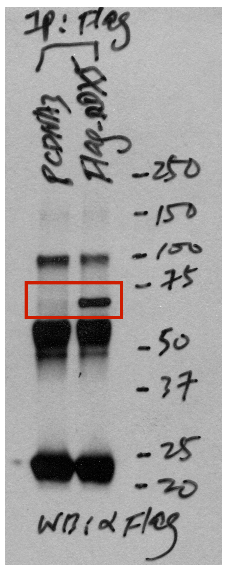

Figure 6A,
right, upper panel

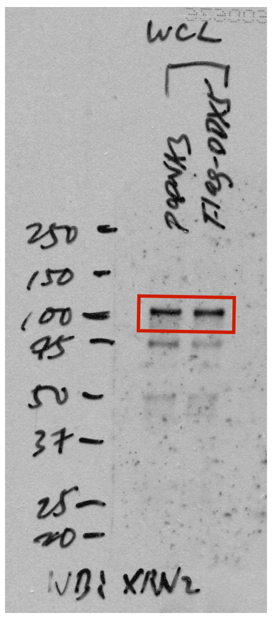

Figure 6A,
left, lower panel

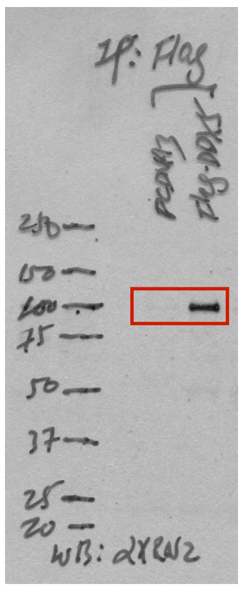

Figure 6A,
right, lower panel

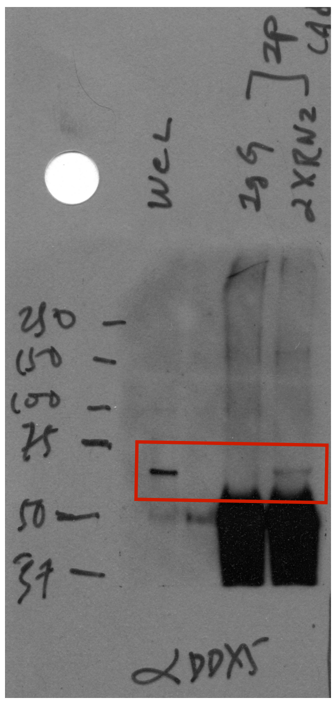

Figure 6B,
upper panel

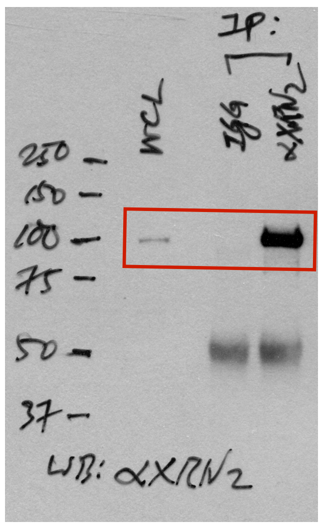

Figure 6B,
lower panel

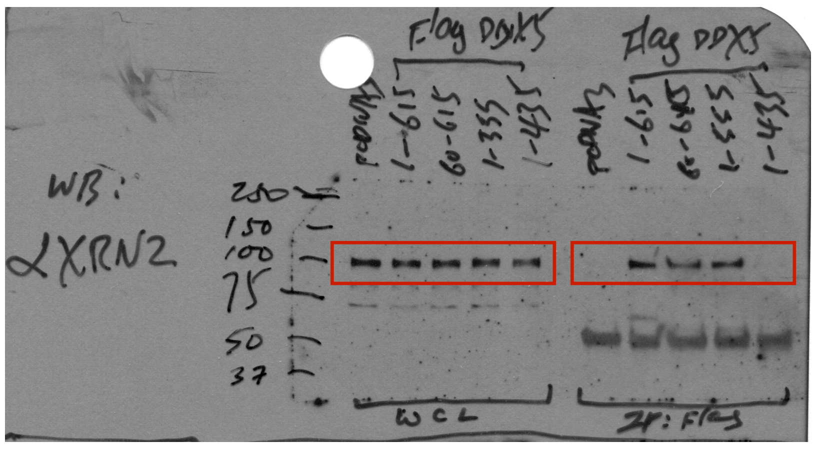

Figure 6C, upper panels

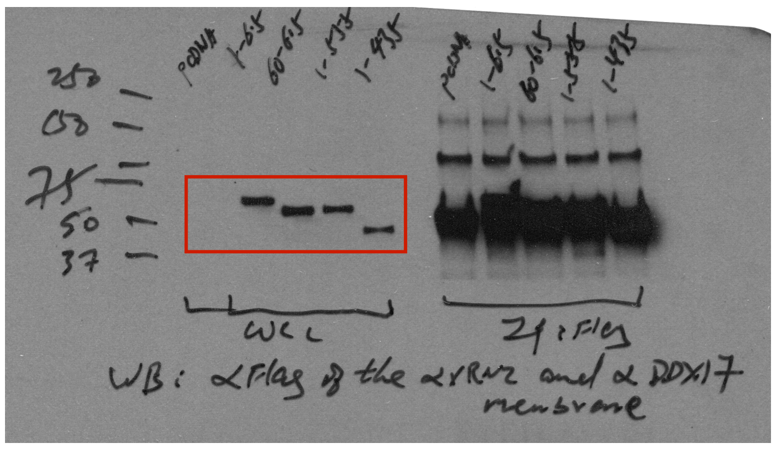

Figure 6C, left, lower panel

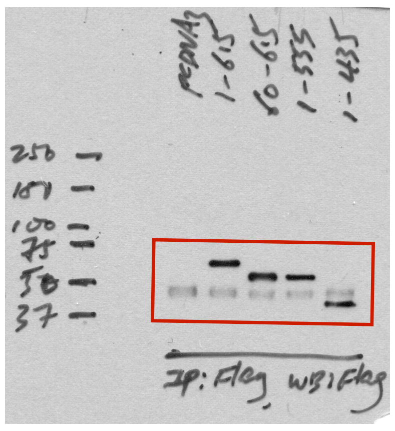

Figure 6C, right, lower panel

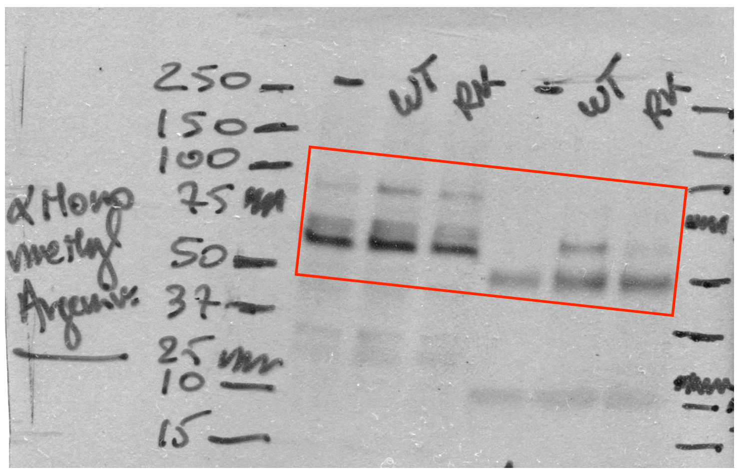

Figure 6D, upper panels

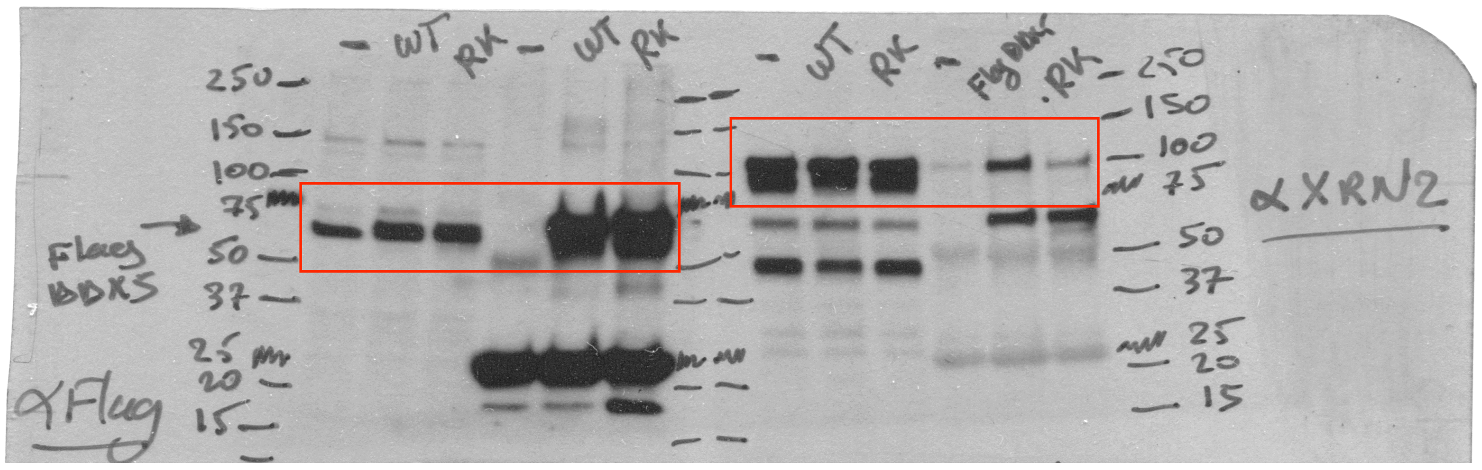

Figure 6C, middle and lower panel
