# Supplementary material for: Arginine methylation of the DDX5 helicase RGG/RG motif by PRMT5 regulates resolution of RNA:DNA hybrids
Source: EMBO J. 2019 Jun 21;38(15):e100986. doi: 10.15252/embj.2018100986 (PMC6669924; doi:10.15252/embj.2018100986)
Supplement: Supplementary file 12 — Source Data for Figure 8 [file EMBJ-38-e100986-s011.pdf]

Mersaoui et al., Figure 8

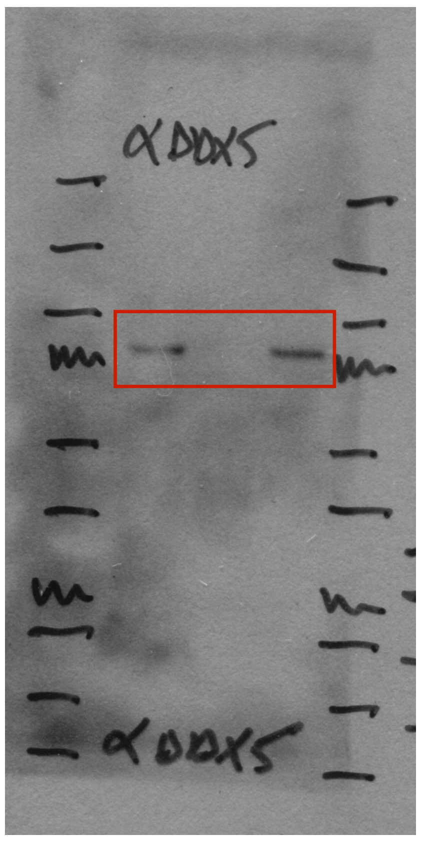

Figure 8C DDX5 Blot

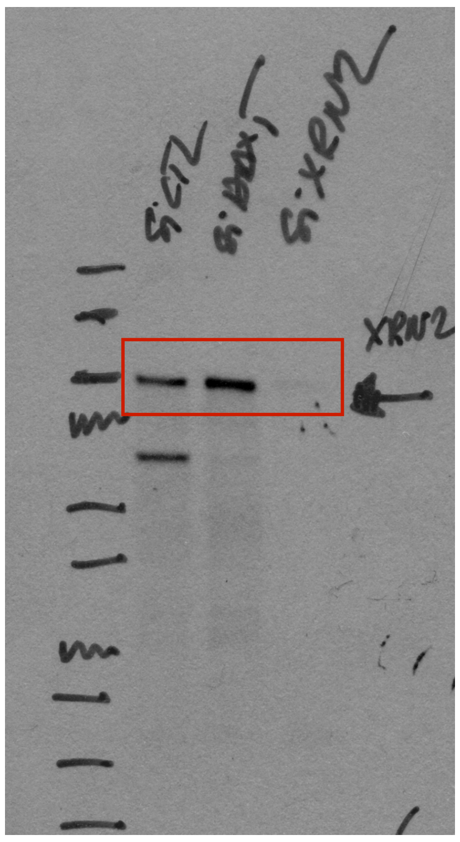

Figure 8C XRN2 Blot

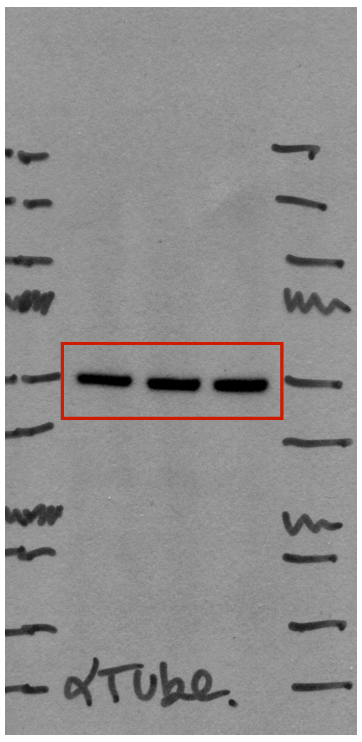

Figure 8C Tubulin Blot

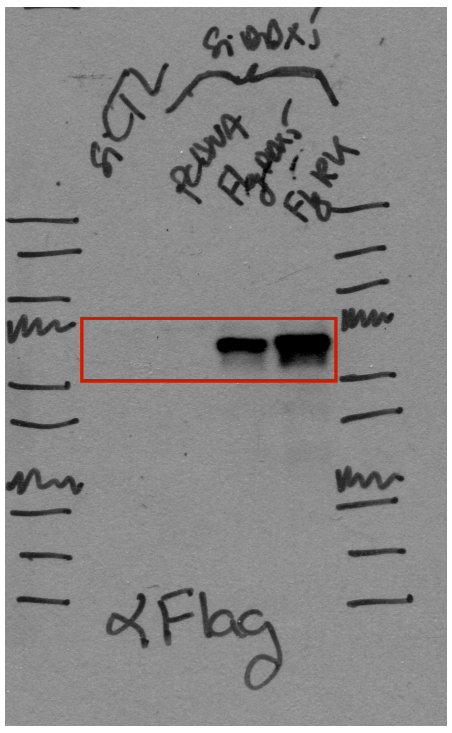

Figure 8D Flag Blot

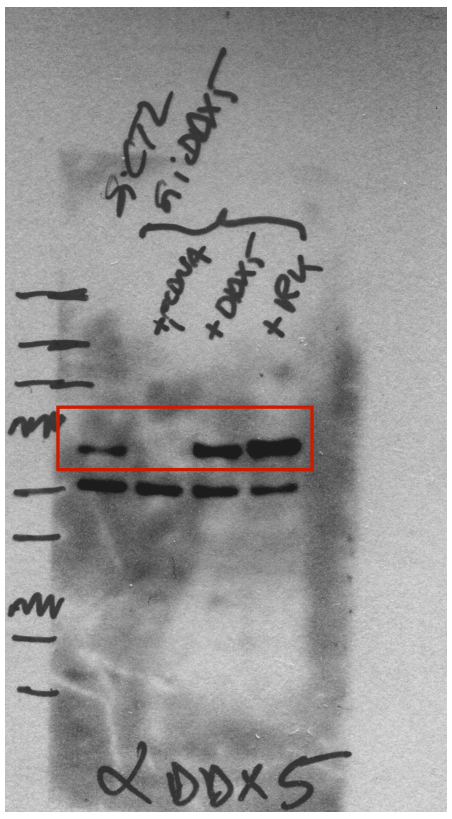

Figure 8D DDX5 Blot

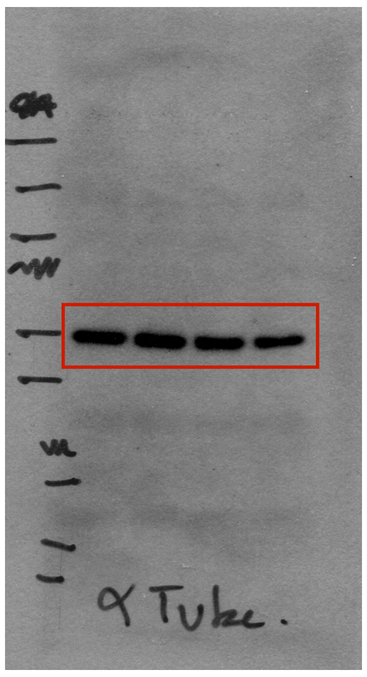

Figure 8D Tubulin Blot

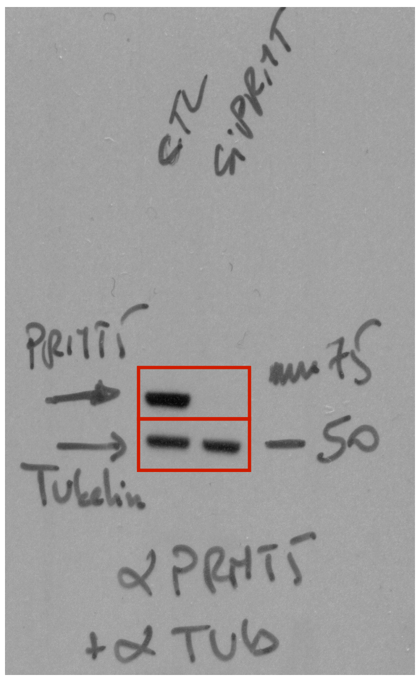

Figure 8E PRMT5 and Tubulin Blot

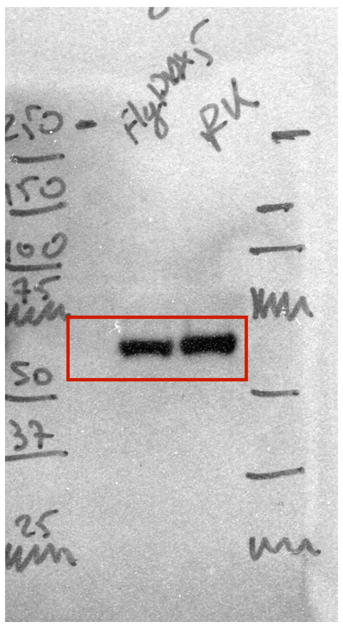

Figure 8F Flag Blot

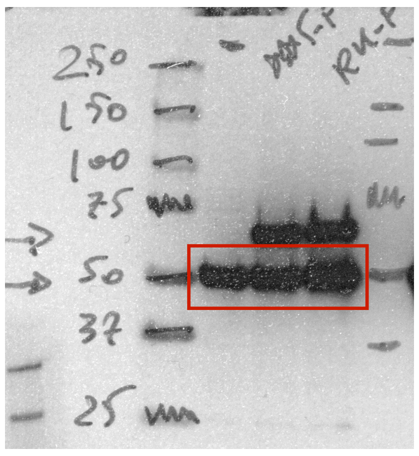

Figure 8F Tubulin Blot
